# Target-driven supramolecular self-assembly for selective amyloid-β photooxygenation against Alzheimer's disease†

**DOI:** 10.1039/d0sc04984k

**Published:** 2020-10-06

**Authors:** Zhenqi Liu, Mengmeng Ma, Dongqin Yu, Jinsong Ren, Xiaogang Qu

**Affiliations:** Laboratory of Chemical Biology, State Key Laboratory of Rare Earth Resource Utilization, Changchun Institute of Applied Chemistry, Chinese Academy of Sciences Changchun Jilin 130022 China xqu@ciac.ac.cn; University of Science and Technology of China Hefei Anhui 230029 China

## Abstract

Photo-oxygenation of β-amyloid (Aβ) has been considered an efficient way to inhibit Aβ aggregation in Alzheimer's disease (AD). However, current photosensitizers cannot simultaneously achieve enhanced blood–brain barrier (BBB) permeability and selective photooxygenation of Aβ, leading to poor therapeutic efficacy, severe off-target toxicity, and substandard bioavailability. Herein, an Aβ target-driven supramolecular self-assembly (PKNPs) with enhanced BBB penetrability and switchable photoactivity is designed and demonstrated to be effective in preventing Aβ aggregation *in vivo*. PKNPs are prepared by the self-assembly of the Aβ-targeting peptide KLVFF and an FDA-approved porphyrin derivative (5-(4-carboxyphenyl)-10,15,20-triphenylporphyrin). Due to the photothermal effect of PKNPs, the BBB permeability of PKNPs under irradiation is 8.5-fold higher than that of porphyrin alone. Moreover, upon selective interaction with Aβ, PKNPs undergo morphological change from the spherical to the amorphous form, resulting in a smart transformation from photothermal activity to photodynamic activity. Consequently, the disassembled PKNPs can selectively oxygenate Aβ without affecting off-target proteins (insulin, bovine serum albumin, and human serum albumin). The well-designed PKNPs exhibit not only improved BBB permeability but also highly selective Aβ photooxygenation. Furthermore, *in vivo* experiments demonstrate that PKNPs can alleviate Aβ-induced neurotoxicity and prolong the life span of the commonly used AD transgenic *Caenorhabditis elegans* CL2006. Our work may open a new path for using supramolecular self-assemblies as switchable phototheranostics for the selective and effective prevention of Aβ aggregation and related neurotoxicity in AD.

## Abstract 2

Photo-oxygenation of β-amyloid (Aβ) has been considered an efficient way to inhibit Aβ aggregation in Alzheimer's disease (AD). We present the first example of Aβ-responsive photodynamic therapy to treatment of AD by using PKNPs self-assemblies.

## Graphical abstract

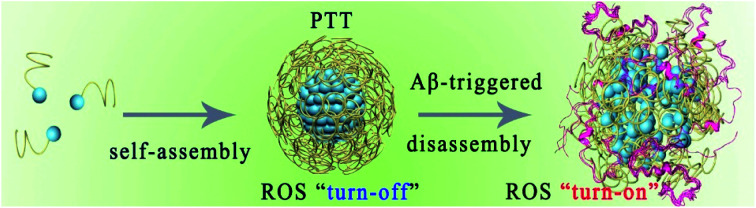

## Introduction

Alzheimer's disease (AD), the most prevalent type of dementia, affects more than 50 million people worldwide. Even worse, with the aging of the population, the number of cases of AD will increase rapidly. Increasing evidence has suggested that the aggregation of amyloid-β peptides (Aβ) is a critical step towards AD pathogenesis.^1^ Accordingly, prevention of Aβ aggregation has been sought as a promising strategy to treat AD.^2,3^ Recently, photo-oxygenation of Aβ has been used for the suppression of Aβ aggregation with unique merits of low invasiveness, high selectivity, and spatiotemporal controllability.^4–6^ Until now, numerous molecular photosensitizers (such as porphyrins,^7^ riboflavin,^8^ and thioflavin T^9,10^) have been reported for the inhibition of Aβ aggregation by photo-oxygenation of Aβ, but none have achieved satisfactory therapeutic effects. The lack of efficacy is mainly attributed to the blood–brain barrier (BBB) with well-structured and dense paracellular tight junctions, which routinely impedes the entry of most therapeutic drugs into the central nervous system (CNS).^11,12^ In addition, these molecular photosensitizers also tend to aggregate and/or suffer from rapid elimination in the body,^13^ further resulting in a decrease in photo-oxygenation efficiency. Most recently, several photoactive nanomaterials with unique BBB penetration ability^14–16^ and physicochemical stability have been developed as promising alternatives to molecular photosensitizers.^17–19^ However, these nanoscale photosensitizers can cause unwanted off-target oxidative damage to healthy tissues due to the reactive oxygen species (ROS) generated under illumination.^6,10^ Hence, development of a novel photodynamic therapy (PDT) strategy with improved brain bioavailability and controllable ROS generation is highly desired.

In nature, the self-assembly of biomolecules into complicated and functionalized units utilizing multiple noncovalent interactions, including electrostatic, hydrophobic, π–π, and coordination interactions, affords a rationale to construct nanostructures.^20–22^ These reversible, controllable, and dynamic noncovalent interactions allow these self-assembled systems of biomolecules to adapt well to the physiological environment to fully optimize their biological functions.^23–26^ For example, the specific interaction among macromolecules causes a conformational change in the partner macromolecule to activate one of the binding partners to trigger a biological cascade.^27^ In particular, low molecular weight peptides exhibit outstanding advantages (such as inherent biocompatibility, potential biodegradability, structural programmability, and easy preparation) compared to other existing self-assembly motifs.^28^ Herein, a peptide-based porphyrin supramolecular self-assembly (PKNPs) with Aβ-responsive structural transformation was designed for selective photooxygenation of Aβ.

**Scheme 1:**
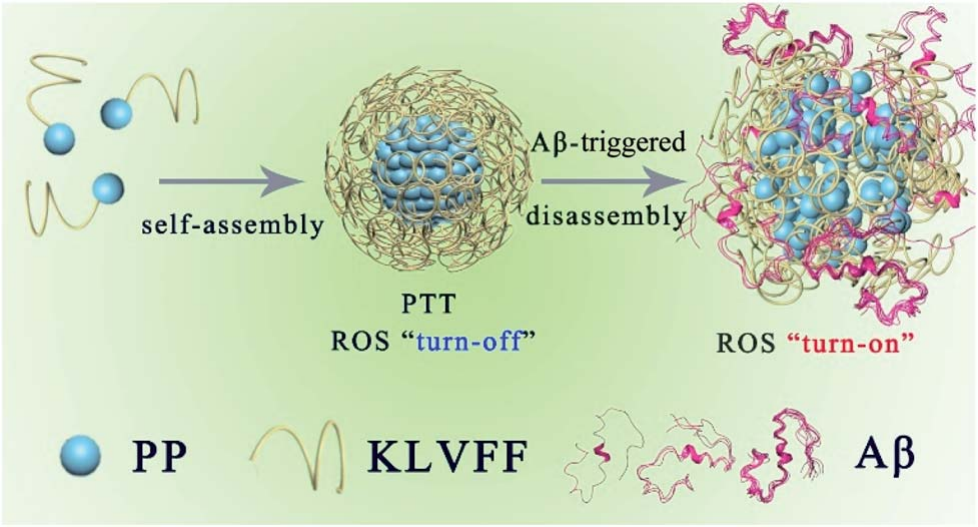
Schematic illustration of the self-assembly process and Aβ-triggered disassembly process of PKNPs.

The PKNPs are prepared by the self-assembly of the US food and drug administration (FDA)-approved porphyrin derivative photosensitizer^29^ (5-(4-carboxyphenyl)-10,15,20-triphenylporphyrin, PP) and Aβ-targeting peptide KLVFF.^30,31^ Porphyrin is selected as a photosensitizer due to its superior optical and electronic properties.^13,32,33^ Besides, porphyrin with intrinsically hydrophobic characteristics could function as a building block in the construction of supramolecular nanostructures. In the study, hydrophobic interactions and π–π stacking interactions facilitate porphyrin–peptide conjugate (PP-KLVFF) self-assembly into spherical nanostructured PKNPs and inhibit their fluorescence emission and ROS generation. Therefore, unsurprisingly, PKNPs exhibit an excellent photothermal effect under illumination, which is conducive to increasing their BBB permeability.^34,49^ More importantly, PKNPs permit disassembly upon specific interaction with Aβ, leading to smart transformation from photothermal activity to photodynamic activity.^35,36^ As a consequence, PKNPs achieve selective photooxygenation of Aβ without affecting non-specific proteins (insulin, bovine serum albumin (BSA), and human serum albumin (HSA)). In this way, PKNPs can realize both improved BBB permeability and highly selective photooxygenation of Aβ by the transformation of morphology (Scheme 1). Furthermore, PKNPs show no obvious toxicity both in rat pheochromocytoma (PC-12) cells and N2 wild-type strain worms. To the best of our knowledge, the use of a supramolecular self-assembly as a novel photosensitizer for activable PDT against AD has not been reported.

## Results and discussion

**Fig. 1:**
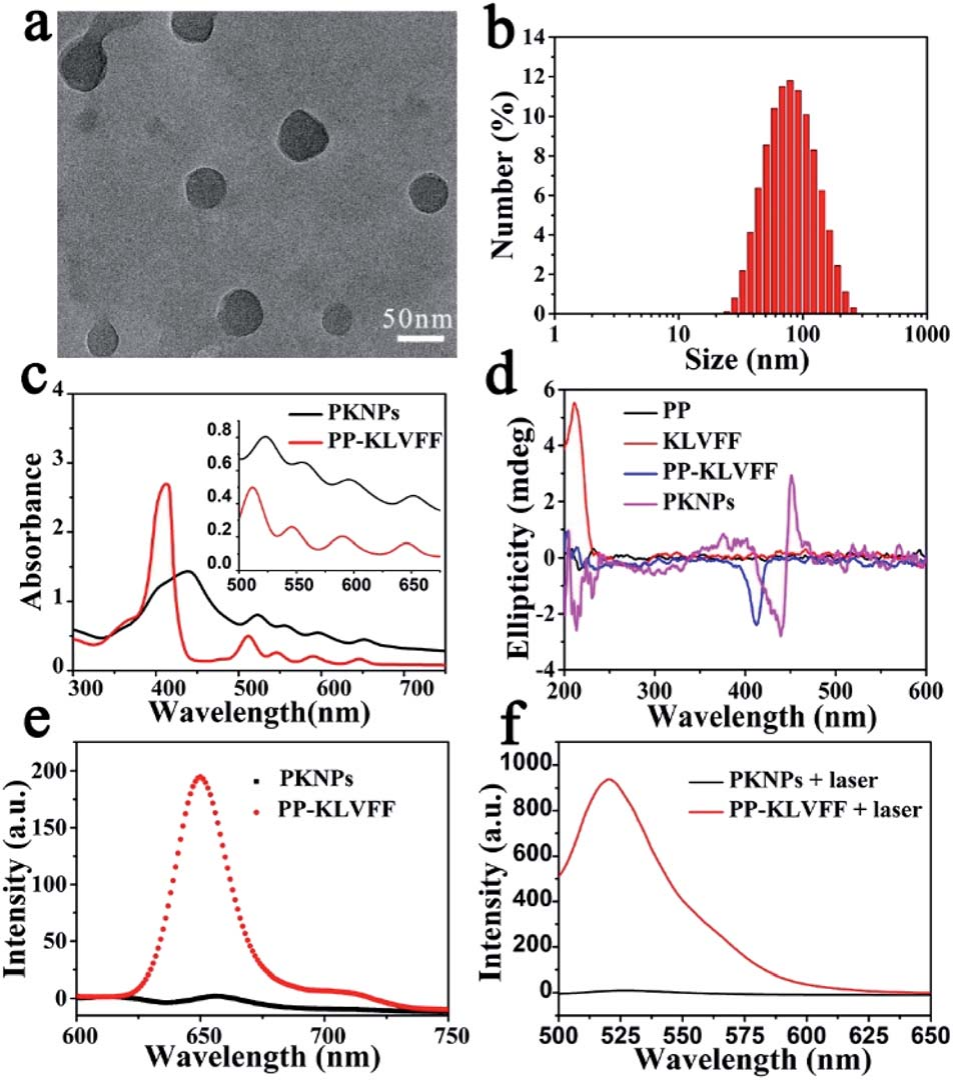
Self-assembly of PP-KLVFF into PKNPs. (a) TEM image of PKNPs. (b) DLS histogram of PKNPs. (c) UV-vis absorption spectra of PP-KLVFF and PKNPs. The inset shows the amplified absorption spectra of Q bands. (d) CD spectra of PP, KLVFF, PP-KLVFF, and PKNPs. (e) Fluorescence emission spectra (*λ*_ex_: 450 nm) of PKNPs and PP-KLVFF. (f) ROS generation by PKNPs and PP-KLVFF, using DCFH-DA (10 μM) as a probe (*λ*_ex_: 488 nm).

KLVFF, a pentapeptide known to specifically target Aβ,^37^ was conjugated to hydrophobic PP to synthesize PP-KLVFF. The resulting PP-KLVFF was verified by ^1^H nuclear magnetic resonance (^1^H NMR, 600 MHz), Fourier transform infrared (FTIR) spectroscopy, mass spectrometry (MS), and high performance liquid chromatography (HPLC) (Fig. S1–S4†). Due to the amphiphilicity, PP-KLVFF could spontaneously assemble into nanostructured PKNPs in DMSO/H_2_O (1 : 9, v/v) solution. Transmission electron microscopy (TEM) images showed that PKNPs possessed a spherical morphology (Fig. 1a). The dynamic light scattering (DLS) histogram of PKNPs revealed an average diameter of 70 nm (Fig. 1b). In addition, a distinct bathochromic shift and broadening of Soret and Q bands in the absorption spectra of PKNPs were observed (Fig. 1c), which could be ascribed to the face-to-face stacking of PP.^33^ The corresponding shift for PKNPs was further investigated by circular dichroism (CD) spectroscopy. As shown in Fig. 1d, a distinct red-shift of the Soret band region in the CD spectrum of PKNPs was observed, which implied strong intermolecular interaction between PP molecules.^38,39^ As shown in Fig. 1e and f, the fluorescence emission and ROS generation of PKNPs were completely quenched, implying that porphyrin molecules were aggregated in aqueous solution. These results together confirmed the successful construction of PKNPs. More importantly, PKNPs had high colloidal stability in water, Dulbecco's modified Eagle's medium (DMEM), and phosphate buffered saline (PBS) (Fig. S5†), showing great potential for biological applications.

Theoretically, photosensitizers are activated from the ground state (S_0_) to the excited state (S_1_) after photoexcitation. However, the high-energy S_1_ is not stable and reverts to the ground state mainly through three processes, including radiative emission (*i.e.*, fluorescence), intersystem crossing (*i.e.*, ROS generation), and vibrational relaxation (*i.e.*, heat).^40^ Interestingly, the aggregation driven by π–π stacking and hydrophobic interactions completely quenched the fluorescence emission (Fig. 1e) and blocked ROS generation (Fig. 1f) of PKNPs, which implied that the light energy absorbed by PKNPs could be transformed into heat.^41,42^ As shown in Fig. S6,† the temperature of the PKNP solution increased monotonically with radiation time, laser intensity, and PKNP concentration, demonstrating the excellent photothermal properties of PKNPs. Meanwhile, PKNPs also exhibited good photothermal stability (Fig. S7†).

**Fig. 2:**
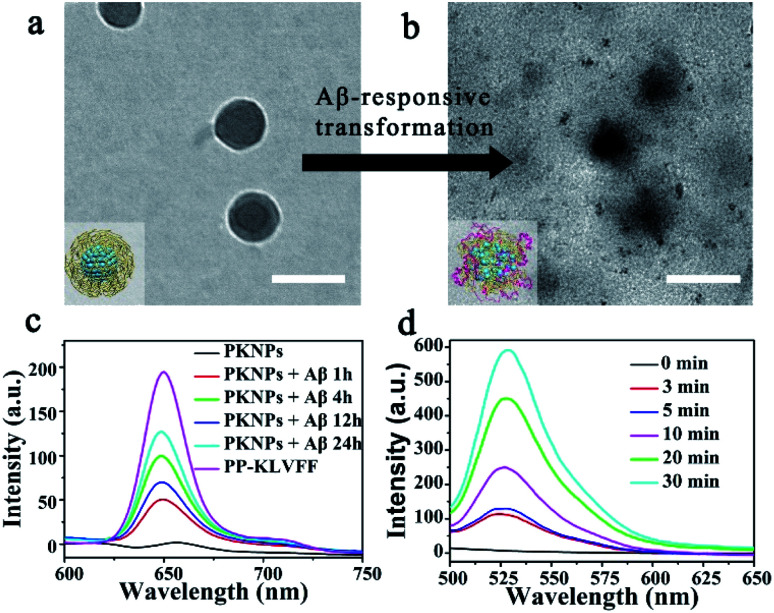
Switchable photoactivity of PKNPs based on the Aβ-driven disassembly. Scale bars: 100 nm. (a) Morphology of integrated PKNPs before adding Aβ. (b) Morphology of dissociated PKNPs after adding Aβ. (c) Fluorescence spectra of PP-KLVFF, PKNPs and PKNPs (0.2 mg mL^−1^) incubated with Aβ (30 μM) for different times. (d) ROS generation by PKNPs (0.2 mg mL^−1^) co-incubated with Aβ (30 μM), using DCFH-DA (10 μM) as the probe (*λ*_ex_: 488 nm).

Since the self-assembly is based on the synergy of noncovalent interactions, it is easily subject to environmental variations.^36,43–45^ Considering the high binding affinity of Aβ with KLVFF, Aβ deposition in the brain of AD patients could trigger the disassembly of PKNPs. To test this hypothesis, the morphology change of PKNPs was first detected by TEM. As shown in Fig. 2a and b, the morphology of PKNPs changed from regular solid spheres to amorphous and fluffy particles upon the addition of Aβ. This result suggested that Aβ could induce PKNP disassembly, which was attributed to the fact that this Aβ binding event disrupted the hydrophilic–lipophilic balance (HLB) of PKNPs.^27,30^ Moreover, the disassembly of PKNPs was further verified from the fluorescence spectra. As depicted in Fig. 2c, in the absence of Aβ, almost no fluorescence emission of PKNPs was observed. However, with the addition of Aβ, the fluorescence intensity significantly increased, suggesting that Aβ disrupted the nanostructure of PKNPs. Subsequently, we explored whether this dissociation process was selectively driven by Aβ. As shown in Fig. S8,† there is no obvious fluorescence increase after PKNPs were incubated with five unrelated proteins, including BSA, HSA, hemoglobin, ferritin, and lysozyme. These results confirmed that specific interactions between Aβ and KLVFF could trigger disassembly of the nanostructure and fluorescence recovery of PKNPs.

The disassembly of the nanostructure and fluorescence recovery of PKNPs triggered by Aβ implies activation of ROS production. Then the ROS generation ability of PKNPs triggered by Aβ was evaluated using 2,7-dichlorofluorescin diacetate (DCFH-DA).^46^ Upon 450 nm laser irradiation, almost no green fluorescence emission was observed in the absence of Aβ (Fig. S9†). Nevertheless, green fluorescence emission was detected upon addition of Aβ (Fig. 2d). In contrast, no fluorescence emission was observed in the presence of various other proteins (Fig. S10†). These results demonstrated that ROS generation was only triggered by Aβ, which highlighted the potential of activable PDT for AD with high selectivity and minimal side effects.

**Fig. 3:**
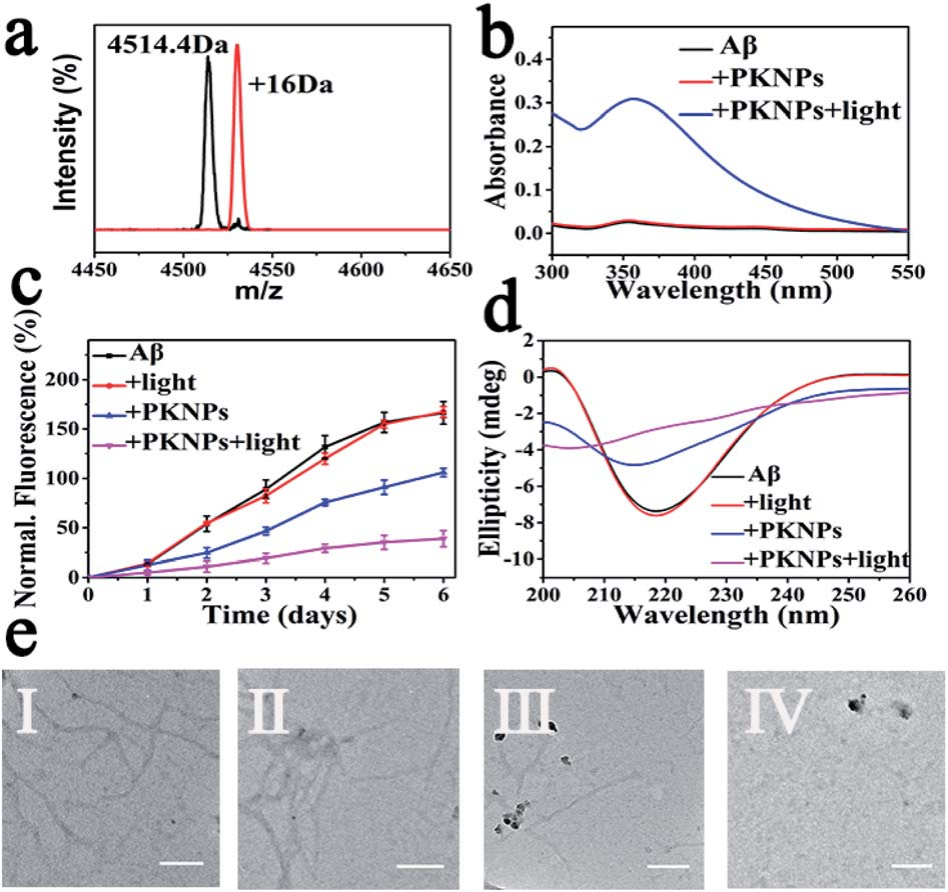
(a) The mass spectra of Aβ_42_ and Aβ_42_ oxidized by PKNPs under laser excitation. (b) DNPH assay. (c) ThT fluorescence assay. (d) CD spectra of Aβ after various treatments. (e) TEM images of different Aβ samples: (I) Aβ, (II) Aβ + light, (III) Aβ + PKNPs, and (IV) Aβ + PKNPs + light. Scale bars are 200 nm.

Next, selective photooxygenation of Aβ was monitored by matrix-assisted laser desorption/ionization time of flight mass spectrometry (MS). As described in Fig. 3a, a +16 Da modification was detected when Aβ was co-incubated with PKNPs and exposed to 450 nm laser irradiation, implying that Aβ was strongly oxidized by PKNPs. In contrast, PKNPs could not oxidize insulin under identical reaction conditions (Fig. S11†). These outcomes confirmed that PKNPs exhibited highly specific photooxygenation of Aβ. Furthermore, the photooxygenation of Aβ was investigated by using 2,4-dinitrophenylhydrazine (DNPH), a sensitive reagent for carbonyl groups of protein samples.^7,47^ As shown in Fig. 3b and S12,† only the carbonyl modification in Aβ samples prominently increased after pre-incubation of Aβ, BSA, and HSA with the PKNPs, respectively. These results verified the selective photooxygenation of Aβ by PKNPs.

Since Aβ was oxidized by PKNPs, we explored whether PKNPs could suppress Aβ aggregation by thioflavin T (ThT) assay. As shown in Fig. 3c, ThT fluorescence was significantly increased when fresh Aβ monomers were incubated at 37 °C for 6 days, indicating the formation of Aβ aggregates. Nevertheless, ThT fluorescence was barely changed when Aβ monomers were incubated with PKNPs and irradiated with 450 nm light. These results suggested that photooxygenation could strongly inhibit Aβ aggregation. Moreover, under dark conditions, PKNPs also suppressed Aβ aggregation to some extent because of their intrinsic ability to inhibit Aβ aggregation of KLVFF. Subsequently, the corresponding morphology change of Aβ was monitored by TEM after various treatments (Fig. 3e). Large branched fibrils were observed in the control group of Aβ alone. However, for photooxygenated Aβ, almost no hundred-nanometer long Aβ fibrils appeared, indicating that photooxygenation of Aβ could remarkably inhibit their aggregation. The above results were also substantiated by CD spectroscopy (Fig. 3d).

Encouraged by the strong inhibition ability of PKNPs against Aβ aggregation, we next investigated whether PKNPs could ameliorate Aβ-mediated cytotoxicity by MTT (3-(4,5-dimethylthiazol-2-yl)-2,5-diphenyltetrazolium bromide) assay. As shown in Fig. S13,† the cell viability in the photooxygenation group was prominently higher than that without laser irradiation. These results demonstrated that PKNPs could effectively attenuate Aβ-induced cytotoxicity through photo-oxygenation of Aβ. In addition, PKNPs had negligible cytotoxicity towards PC-12 cells with a PKNP concentration of up to 0.5 mg mL^−1^, indicating their good biocompatibility (Fig. S14†).

The rigid BBB is the main obstacle that impedes the entry of most drugs into the brain.^11,48^ Recently, nanoparticles with intrinsic BBB-penetrability have been developed for neurodegenerative disease treatment.^12,14,16^ Therefore, we inferred that solid spherical nanostructured PKNPs exhibited great potential for traversing the BBB. Moreover, considering the photothermal effect of PKNPs, the BBB permeability of PKNPs could be improved under NIR irradiation.^34,49^ Thus, the ability of PKNPs to penetrate the BBB was studied by using *in vitro* BBB models based on a murine brain endothelial cell line (bEnd.3).^50,51^ As described in Fig. S15,† the penetration efficiency of PP and PKNPs across the BBB model was calculated to be 2.12% and 3.09%, respectively. Upon 638 nm laser irradiation, the penetration efficiency of PKNPs was further increased and reached 17.93%, proving that the BBB permeability of PKNPs could be enhanced by the photothermal effect. It should be noted that the transepithelial electrical resistance (TEER) values were stable throughout the experiment, suggesting that the mild photothermal effect did not impact BBB integrity.

**Fig. 4:**
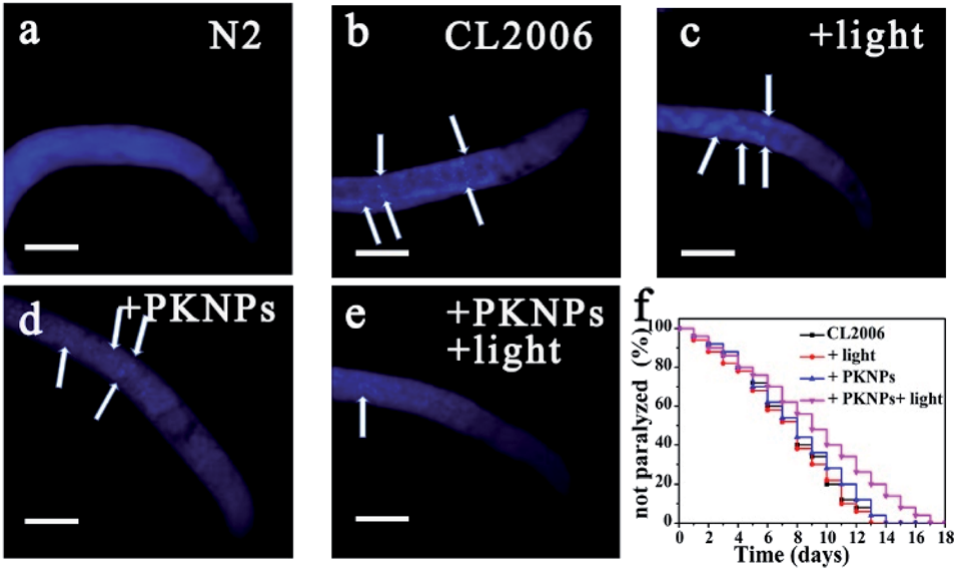
PKNPs decreased Aβ deposition and relieved Aβ-triggered paralysis and motility impairment of CL 2006 nematodes. Scale bars are 20 μm. (a–e) ThS-staining images of Aβ deposits. The arrows indicate Aβ plaques. (a) Bristol N2 strains. CL2006 incubated on the nematode growth medium (NGM) (b) alone, (c) exposed to laser irradiation, (d) with PKNPs (0.2 mg mL^−1^), and (e) in the presence of PKNPs under irradiation. (f) Survival curves of CL2006 worms under different conditions.

The AD transgenic strain *C. elegans* CL2006, a widely used AD model, is characterized by Aβ peptides expressed in muscle cells.^52^ Thioflavin S (ThS) staining was performed to evaluate the effect of PKNPs on the Aβ deposits in the CL2006 worms (Fig. 4b–e).^53^ Compared to the N2 wild-type strain (Fig. 4a), the levels of aggregates were apparently increased in the untreated CL2006 strain. However, Aβ deposits were significantly decreased after feeding with PKNPs and subsequent exposure to a 450 nm laser (Fig. 4e). As a consequence, PKNPs could relieve Aβ-caused motility impairment and paralysis of CL2006 worms and significantly extend the life span of CL2006 nematodes under 450 nm light irradiation (Fig. S16† and 4f). In contrast, neither PKNPs nor 450 nm light alone decreased Aβ deposition and delayed the paralysis of worms. These results demonstrated that PKNPs could reduce the Aβ burden in the CL2006 strain under 450 nm laser irradiation.

## Conclusion

In summary, we present the first example of Aβ-responsive activable PDT for treatment of AD by using nanostructured PKNP self-assemblies. Due to the photothermal effect of PKNPs, the BBB permeability of PKNPs under irradiation is 8.5 and 5.8 times higher compared to that of porphyrin and PKNPs alone, respectively. Moreover, attributed to the noncovalent interactions of the Aβ-targeting peptide KLVFF and PP, the spherical PKNPs show their ability for Aβ-driven disassembly and thus achieve Aβ-specific triggered ROS generation. As a result, PKNPs can act as an activable photosensitizer for selective photooxygenation of Aβ and inhibition of aggregation without off-target side effects. Furthermore, *in vivo* studies indicate that PKNPs can attenuate Aβ-mediated toxicity and extend the life span of CL2006 worms. Taken together, our designed switchable supramolecular self-assembly can realize selective and effective prevention of Aβ aggregation and related neurotoxicity in an AD model.

## Conflicts of interest

There are no conflicts to declare.

## Supplementary Material

SC-011-D0SC04984K-s001

## References

[cit1] Hardy J., Selkoe D. J. (2002). Science.

[cit2] Ke P. C., Pilkington E. H., Sun Y., Javed I., Kakinen A., Peng G., Ding F., Davis T. P. (2020). Adv. Mater..

[cit3] Eisele Y. S., Monteiro C., Fearns C., Encalada S. E., Wiseman R. L., Powers E. T., Kelly J. W. (2015). Nat. Rev. Drug Discovery.

[cit4] Bondia P., Torra J., Tone C. M., Sawazaki T., Del Valle A., Sot B., Nonell S., Kanai M., Sohma Y., Flors C. (2020). J. Am. Chem. Soc..

[cit5] Ahn M., Lee B. I., Chia S., Habchi J., Kumita J. R., Vendruscolo M., Dobson C. M., Park C. B. (2019). Chem. Commun..

[cit6] Lee B. I., Chung Y. J., Park C. B. (2019). Biomaterials.

[cit7] Lee B. I., Lee S., Suh Y. S., Lee J. S., Kim A. K., Kwon O. Y., Yu K., Park C. B. (2015). Angew. Chem., Int. Ed..

[cit8] Taniguchi A., Sasaki D., Shiohara A., Iwatsubo T., Tomita T., Sohma Y., Kanai M. (2014). Angew. Chem., Int. Ed..

[cit9] Yagi H., Ozawa D., Sakurai K., Kawakami T., Kuyama H., Nishimura O., Shimanouchi T., Kuboi R., Naiki H., Goto Y. (2010). J. Biol. Chem..

[cit10] Taniguchi A., Shimizu Y., Oisaki K., Sohma Y., Kanai M. (2016). Nat. Chem..

[cit11] Ali I. U., Chen X. (2015). ACS Nano.

[cit12] Furtado D., Bjornmalm M., Ayton S., Bush A. I., Kempe K., Caruso F. (2018). Adv. Mater..

[cit13] Rajora M. A., Lou J. W. H., Zheng G. (2017). Chem. Soc. Rev..

[cit14] Xie J., Shen Z., Anraku Y., Kataoka K., Chen X. (2019). Biomaterials.

[cit15] Zhang L., Liu X. g., Liu D. q., Yu X. l., Zhang L. x., Zhu J., Lu S., Liu R. t. (2020). Adv. Funct. Mater..

[cit16] Mukherjee S., Madamsetty V. S., Bhattacharya D., Chowdhury S. R., Paul M. K., Mukherjee A. (2020). Adv. Funct. Mater..

[cit17] Kuk S., Lee B. I., Lee J. S., Park C. B. (2017). Small.

[cit18] Li Y., Du Z., Liu X., Ma M., Yu D., Lu Y., Ren J., Qu X. (2019). Small.

[cit19] Chung Y. J., Lee B. I., Park C. B. (2019). Nanoscale.

[cit20] Wang J., Liu K., Xing R., Yan X. (2016). Chem. Soc. Rev..

[cit21] Fan Z., Sun L., Huang Y., Wang Y., Zhang M. (2016). Nat. Nanotechnol..

[cit22] Yang P. P., Zhang K., He P. P., Fan Y., Gao X. J., Gao X., Chen Z. M., Hou D. Y., Li Y., Yi Y., Cheng D. B., Zhang J. P., Shi L., Zhang X. Z., Wang L., Wang H. (2020). Sci. Adv..

[cit23] Sun H., Li Y., Yu S., Liu J. (2020). Front. Bioeng. Biotechnol..

[cit24] Yao Q., Lin F., Fan X., Wang Y., Liu Y., Liu Z., Jiang X., Chen P. R., Gao Y. (2018). Nat. Commun..

[cit25] Nguyen M. M., Carlini A. S., Chien M. P., Sonnenberg S., Luo C., Braden R. L., Osborn K. G., Li Y., Gianneschi N. C., Christman K. L. (2015). Adv. Mater..

[cit26] Wijnands S. P. W., Engelen W., Lafleur R. P. M., Meijer E. W., Merkx M. (2018). Nat. Commun..

[cit27] Molla M. R., Prasad P., Thayumanavan S. (2015). J. Am. Chem. Soc..

[cit28] Qi G. B., Gao Y. J., Wang L., Wang H. (2018). Adv. Mater..

[cit29] Vicente M. (2001). Adv. Anticancer Agents Med. Chem..

[cit30] Yang P.-P., Zhao X.-X., Xu A.-P., Wang L., Wang H. (2016). J. Mater. Chem. B.

[cit31] Du Z., Gao N., Wang X., Ren J., Qu X. (2018). Small.

[cit32] Liu K., Xing R., Zou Q., Ma G., Mohwald H., Yan X. (2016). Angew. Chem., Int. Ed..

[cit33] Zou Q., Abbas M., Zhao L., Li S., Shen G., Yan X. (2017). J. Am. Chem. Soc..

[cit34] Chen W., Ouyang J., Yi X., Xu Y., Niu C., Zhang W., Wang L., Sheng J., Deng L., Liu Y. N., Guo S. (2018). Adv. Mater..

[cit35] Li X., Kim C. Y., Lee S., Lee D., Chung H. M., Kim G., Heo S. H., Kim C., Hong K. S., Yoon J. (2017). J. Am. Chem. Soc..

[cit36] Li S., Zhao L., Chang R., Xing R., Yan X. (2019). Chemistry.

[cit37] Pham J. D., Spencer R. K., Chen K. H., Nowick J. S. (2014). J.
Am. Chem. Soc..

[cit38] Ethordevic L., Arcudi F., D'Urso A., Cacioppo M., Micali N., Burgi T., Purrello R., Prato M. (2018). Nat. Commun..

[cit39] Charalambidis G., Georgilis E., Panda M. K., Anson C. E., Powell A. K., Doyle S., Moss D., Jochum T., Horton P. N., Coles S. J., Linares M., Beljonne D., Naubron J. V., Conradt J., Kalt H., Mitraki A., Coutsolelos A. G., Balaban T. S. (2016). Nat. Commun..

[cit40] Zhao L., Liu Y., Xing R., Yan X. (2020). Angew. Chem., Int. Ed..

[cit41] Xi D., Xiao M., Cao J., Zhao L., Xu N., Long S., Fan J., Shao K., Sun W., Yan X., Peng X. (2020). Adv. Mater..

[cit42] Mu X., Lu Y., Wu F., Wei Y., Ma H., Zhao Y., Sun J., Liu S., Zhou X., Li Z. (2020). Adv. Mater..

[cit43] Sun B., Chang R., Cao S., Yuan C., Zhao L., Yang H., Li J., Yan X., van Hest J. (2020). Angew. Chem., Int. Ed..

[cit44] Mizusawa K., Ishida Y., Takaoka Y., Miyagawa M., Tsukiji S., Hamachi I. (2010). J. Am. Chem. Soc..

[cit45] Takaoka Y., Kiminami K., Mizusawa K., Matsuo K., Narazaki M., Matsuda T., Hamachi I. (2011). J. Am. Chem. Soc..

[cit46] Wang Z., Zhang Y., Ju E., Liu Z., Cao F., Chen Z., Ren J., Qu X. (2018). Nat. Commun..

[cit47] Yu D., Guan Y., Bai F., Du Z., Gao N., Ren J., Qu X. (2019). Chemistry.

[cit48] Gopalan D., Pandey A., Udupa N., Mutalik S. (2019). J. Controlled Release.

[cit49] Liu W., Wang W., Dong X., Sun Y. (2020). ACS Appl. Mater. Interfaces.

[cit50] Yin T., Xie W., Sun J., Yang L., Liu J. (2016). ACS Appl. Mater. Interfaces.

[cit51] Guan Y., Li M., Dong K., Gao N., Ren J., Zheng Y., Qu X. (2016). Biomaterials.

[cit52] Du Z., Yu D., Du X., Scott P., Ren J., Qu X. (2019). Chem. Sci..

[cit53] Gao N., Du Z., Guan Y., Dong K., Ren J., Qu X. (2019). J. Am. Chem. Soc..

