# Supplementary material for: Target-driven supramolecular self-assembly for selective amyloid-β photooxygenation against Alzheimer's disease
Source: Chem Sci. 2020 Oct 6;11(40):11003–8. doi: 10.1039/d0sc04984k (PMC8162409; doi:10.1039/d0sc04984k)
Supplement: SC-011-D0SC04984K-s001 [file SC-011-D0SC04984K-s001.pdf]

Electronic Supplementary Material (ESI) for Chemical Science.
This journal is © The Royal Society of Chemistry 2020

Supporting Information

Target-driven Supramolecular Self-assembly for Selective Amyloid-β Photooxygenation Against Alzheimer's Disease

Zhenqi Liu,[a,b] Mengmeng Ma,[a,b] Dongqin Yu,[a,b] Jinsong Ren,[a,b] and Xiaogang Qu[a,b]*

1. Materials

$A\beta_{42}$ peptides were purchased from NovoPep Limited (Shanghai, China). 1,1,1,3,3,3-hexafluoro-2-propanol (HFIP), Dulbecco's modified Eagle's medium (DMEM), and 3-[4,5-dimethylthiazol-2-yl]-2,5-diphenyltetrazolium-bromide (MTT) were received from Sigma-Aldrich. N-hydroxysulfosucnimide sodium (NHS) and 1-[3-(dimethylamino)propyl]-3-ethylcarbodiimide hydrochloride (EDC) were purchased from Alfa Aesar. 2,4-dinitrophenyhydration (DNPH) and guanidine hydrochloride solution were obtained from Macklin. 5-(4-Carboxyphenyl)-10,15,20-triphenylporphyrin (PP) was purchased from Yanshen Technology Co., Ltd (Changchun, China). Other chemicals were purchased from Aladdin. Milli-Q water (18.2 MΩ; Millpore Co. USA) was used throughout all experiment. The high performance liquid chromatography (HPLC) was measured by Ultimate 3000. All of the chemical reagents were analytical grade and directly used without further purification unless otherwise specified.

2. Measurements and characterizations

Ultraviolet-visible spectroscopy measurements were recorded on a Jasco-V550 spectrophotometer. TEM experiments were performed with Philips Tacnai G2 20 S-TWIN microscope. Fluorescence spectroscopy measurements were conducted on JASCO FP-6500 spectrofluorometer. CD spectroscopy measurements were measured on JASCO J-810 spectropolarimeter. MS spectra were recorded on autoflexIII smartbeam MALDI-TOF (Bruker) using sinapic acid as the matrix. Dynamic light scattering (DLS) of the nanoparticles were measured in the Zetasizer Nano (Malvern Instruments, Worchestershire, UK). The laser irradiation experiments were operated by CW diode laser (LSR808NL-2000) with wavelength of 450 nm and 638nm. Chemical structures were drawn using *Kingdraw* software.

3. Synthesis

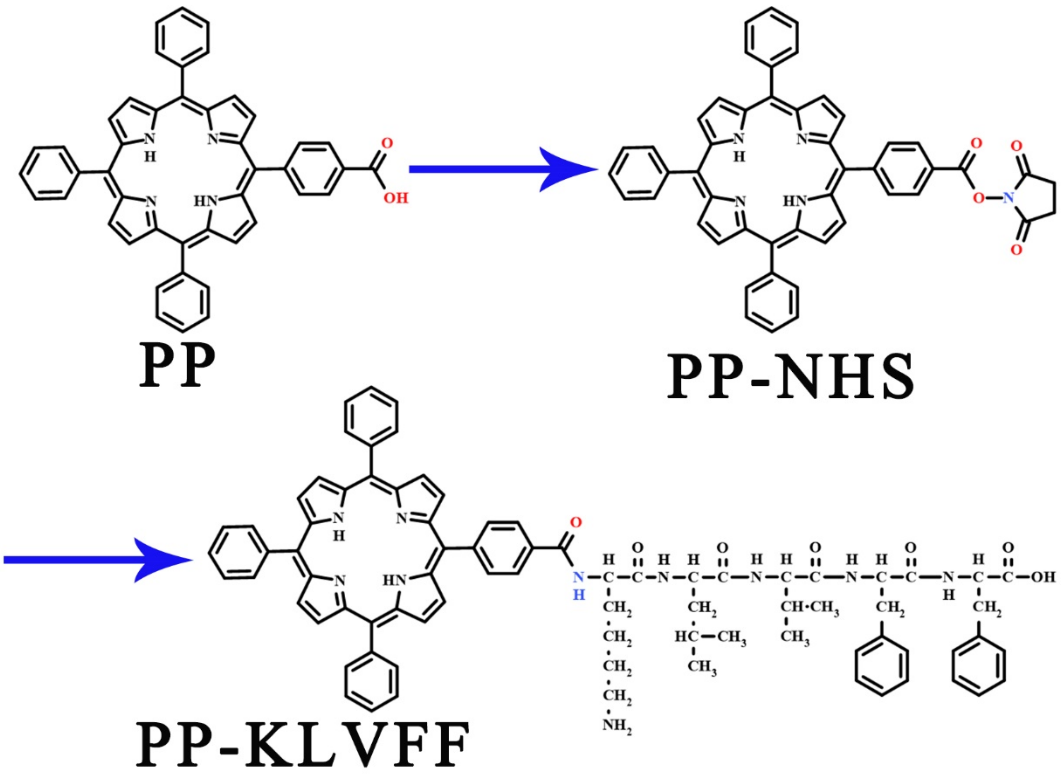

PP-KLVFF 20 mg 5-(4-Carboxyphenyl)-10,15,20-triphenylporphyrin (PP, 0.03mmol), 5.9 mg EDC (0.03mmol), 2.5 mg NHS (0.03mmol) and 1 mL dimethylformamide (DMF) were added in a round bottom flask. The mixture was stirred by a magnetic stirrer for 6 h at room temperature followed by the addition of 2 mL water under stirring. 13.23mg KLVFF (0.02mmol) was dissolved in 0.5 mL distilled water and 0.28 mL triethylamine (TEA) and then added to the mixture. The reaction mixture was stirred for 12 h at room temperature. Then 4 mL distilled water was added to the reaction mixture. After acidification of the solution to pH 3 by HCl, the product was extracted by ethyl acetate (20 mL × 3) through a separating funnel. The combined organic phase was washed by distilled water (20 mL × 3) and evaporated. The crude product was collected and further purified by silica gel column chromatography to determine the product (yield 31%). Finally, the obtained PP-KLVFF was dissolved in DMSO for further use (0.5 mg mL^{-1}).

Preparation of PKNPs 100 μL DMSO solution of PP-KLVFF (0.5 mg mL^{-1}) was mixed with 900 μL pure water, and the obtained dispersive PKNPs were aged for 24 h and dialyzed against water. For dialysis, 1 mL PKNPs were sealed in a dialysis bag (molecular weight cut-off: 3.5 kDa) and immersed in 1 L pure water for 48 h, during which the water was replaced for 3 times. Finally, PKNPs were collected by vacuum freeze-drying .

4. Protein sample preparation

$A\beta_{42}$ was prepared following a previous protocol. At first, Aβ was dissolved in HFIP and stored at -20 °C as the stock solution. Before use, the solvent HFIP was evaporated and Aβ was redissolved in 20 mM Tris buffer (pH 7.4). For the Aβ aggregation, the solution was incubated at 37 °C for 72 hours.

5. *In vitro* ROS generation tests

For a typical test, the PKNPs (0.2 mg mL^{-1}) were mixed with the stock solution of DCFH (10 μM) in PBS buffer (pH=7.4) containing $A\beta_{42}$ (30 μM), which simulated pathological Aβ accumulation in AD brains. Then, the mixture was irradiated by 450 nm laser at a power density of 0.57 W cm^{-2}. Immediately after the irradiation, the solutions were centrifuged, and the fluorescence of the supernatants was measured for the estimation of the produced ROS.

6. Aβ photooxidation

For CD, MS, ThT, and TEM analysis, $A\beta_{42}$ (30 μM) was incubated in the absence or presence of PKNPs (0.2 mg mL^{-1}), either in darkness or laser irradiated (450nm, 0.57 W cm^{-2}) for 30 min. Next, the mixtures were further aged for 72 h, to allow Aβ aggregation.

For the DNPH assay, the Aβ/BSA/HSA solutions (40 mM, 480 μl) in the absence or presence of PKNPs (0.2 mg mL^{-1}) were precipitated with 20% TCA (trichloroacetic acid) solution in ice bath and then collected by centrifuge. Subsequently, 500 μL of 2M HCl containing 10 mM DNPH (2M HCl only for reagent blanks) was added dropwise to the protein pellet, followed by incubation under room temperature for 1h. The samples were precipitated with 20% TCA solution and then washed three times with 1 ml ethanol-ethyl acetate (1:1, v/v) solution. The sample was resuspended in a 6M guanidine hydrochloride solution (in 20 mM potassium phosphate, pH 2.3 adjusted with TCA) at 37 ˚C for 15 min. The absorbance spectrums of the samples were measured using UV-Vis absorbance spectrometer.

7. *In vitro* Photothermal activity

Aqueous solution (1.0 mL) of PKNPs (0.2 mg mL^{-1}) was put in a quartz cuvette and irradiated with a 638 nm laser for 8 min. Pure water was used as a control group. A thermocouple probe with a digital thermometer was used to measure the temperature every 60 s.

8. Cell culture

PC12 cells (rat pheochromocytoma) were cultured in Dulbecco's Modified Eagle Medium (DMEM) supplemented with 5% fetal bovine serum, 10% horse serum in the CO_2 (5%) environment at 37 °C. Cells were placed at a density of 1.0×10^5 cells per well on 96-well plates in fresh medium.

Mouse brain microvessel endothelial (bEnd.3) cells were cultured in DMEM medium supplemented with 10% fetal bovine serum (FBS), 1% streptomycin/penicillin. Cells were kept at 37 °C in a humidified atmosphere with 5% CO_2.

9. Cell viability assays

The cell viability was measured by MTT assays. The PC12 cells were seeded at a density of 10,000 cells cells/100 μL in a 96-well plate. To validate the inhibition of Aβ-mediated cellular toxicity by PKNPs. Aβ monomers (30 μM) were pretreated with or without PKNPs (0.2 mg mL^{-1}), then the samples were added in each well and the cells were further cultured for 12 h. After that, cells were irradiated with laser (450nm, 0.57 W cm^{-2}) for 3 min with 2 min interval and for 15 times. Cells were incubated for another 24 h after the laser excitation. Finally, 20 μL MTT (5 mg/mL) was added into the plate. The culture medium containing MTT was replaced with DMSO (100 μL) after incubation for 4 h. The absorbance (490 nm) was measured using a microplate reader

10. *in vitro* blood–brain barrier (BBB) transportation study

The *in vitro* BBB model was fabricated according the literature reported method. Briefly, bEnd.3 cells were seeded in the upper chamber of transwell plate at the density of 5 × 10^4 cells per well to form a tight monolayer. The medium was changed every other day and the transepithelial electrical resistance (TEER) was measured using a TEER instrument (Word Precision Instruments, Inc. Sarasota, USA). When the TEER reached 150 Ω cm^2, the medium in the upper chamber of each well was replaced with PKNPs (0.2 mg mL^{-1}) dispersed in HEPES-KRH buffer (136 mM NaCl, 0.9 mM $CaCl_2$, 0.5 mM $MgCl_2$, 2.7 mM KCl, 1.5 mM KH_2PO_4, 10 mM NaH_2PO_4, 25 mM glucose and 10 mM HEPES, pH 7.4). Then the cell monolayer was irradiated under 638 nm laser (1.2 W cm^{-2}) for 3 minutes with 3 minutes interval and for 10 times. Meanwhile, cell monolayer without irradiation was also used as control. The concentration of PKNPs in the upper chamber was measured using UV-Vis absorbance spectrometer according to the standard curve. The transport ratio was calculated with the formula: ratio% = (1 - W_n/W) × 100, where W is the concentration of PKNPs of samples added in apical chamber, and W_n is the concentration of PKNPs of samples taken from upper chamber after 24 h.

11. C. elegans experiments

C. elegans strain was fed with E. coli (OP50) and cultured at 20 °C on the nematode growth medium (NGM) after the egg synchronization. Parallel worms were cultured on solid NGM containing PKNPs (0.2 mg mL^{-1}) and then irradiated with laser. For ThS-staining experiments, worms were fixed with 4% paraformaldehyde and then permeabilized with β-mercaptoethanol before immersing in ThS solution (0.125%) for

4 min. For life span experiments, when strains only moved their head or did not move at all even gently touched by a platinum loop, we classified them as paralyzed. Three independent trials were performed.

12. Statistical analysis

All data were expressed in this article as mean resultstandard deviation (SD). All figures shown in this article were obtained from three independent experiments with similar results.

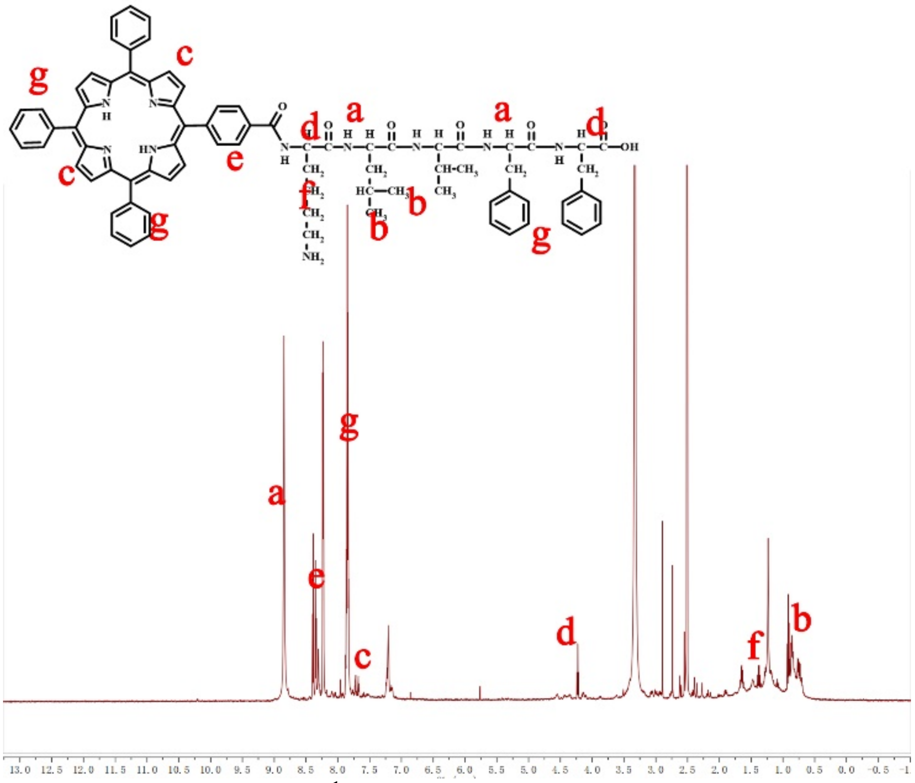

Figure S1. ^{1}H NMR spectra of PP-KLVFF

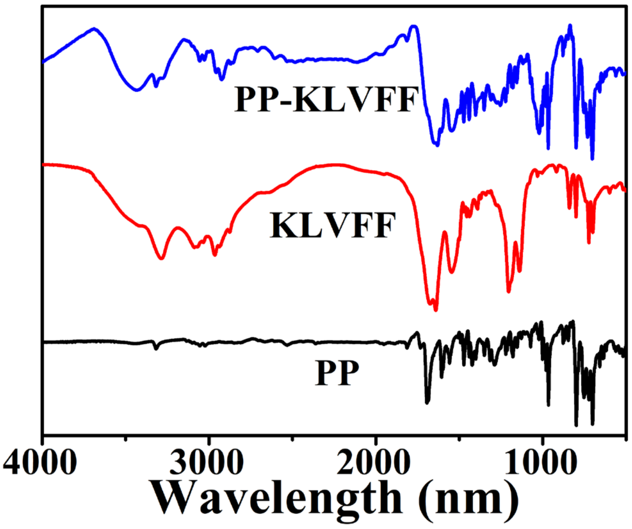

Figure S2. FI-TR spectra of PP, KLVFF, and PP-KLVFF.

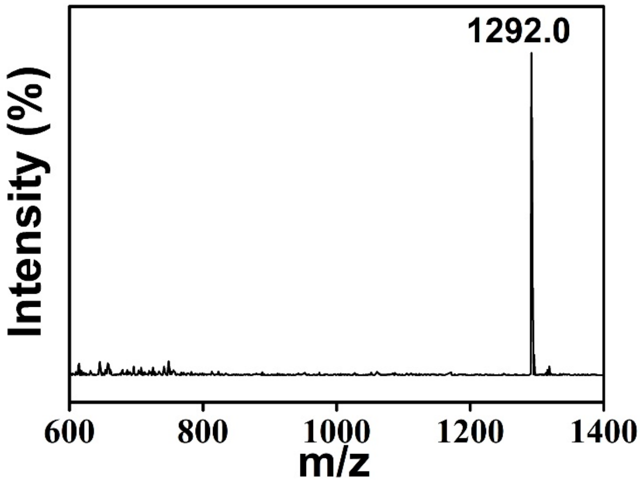

Figure S3. Mass spectra of PP-KLVFF.

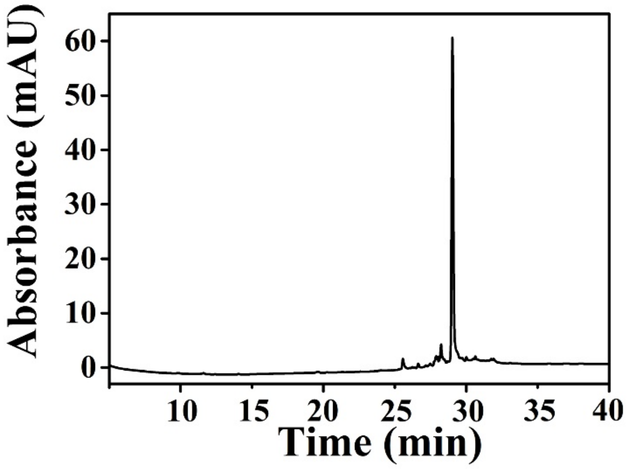

Figure S4. HPLC chromatogram of PP-KLVFF.

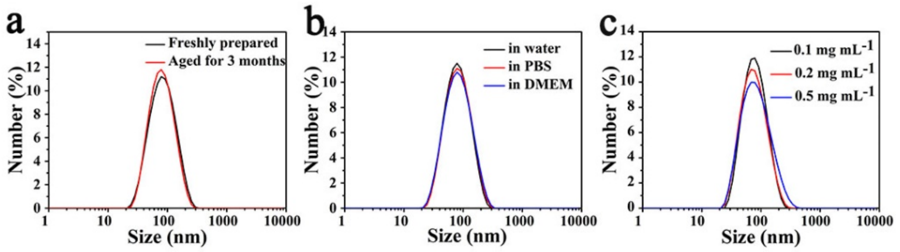

Figure S5. Stability of PKNPs. (a) DLS size profiles of freshly prepared and aged PKNPs. (b) DLS size profiles of PKNPs in water, in PBS and in DMEM. (c) DLS size profiles for various concentrations of PKNPs.

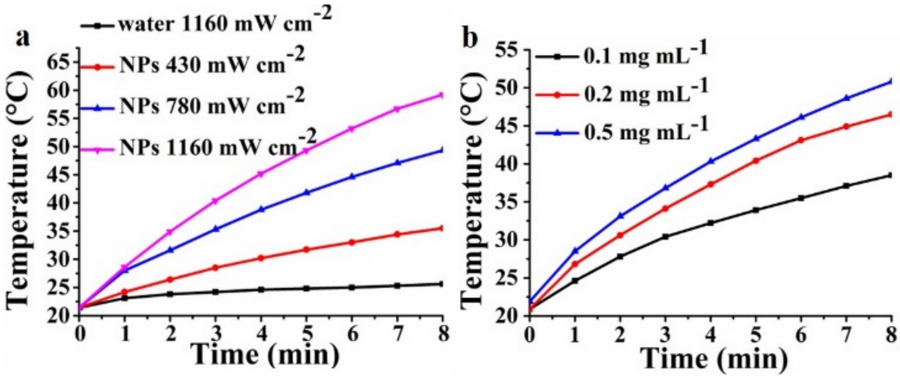

FigureS6. Photothermal properties of PKNPs. (a) Temperature elevation for PKNPs (0.5 mg mL^{-1}) in water in dependence of light intensity. (b) Temperature elevation depends on the concentration of PKNPs under laser irradiation (638nm, 0.78 W cm^{-2}).

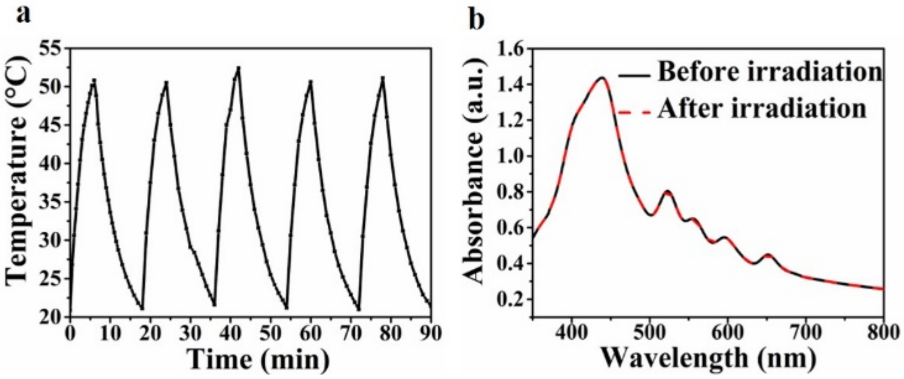

Figure S7. (a) Continuous irradiation-cooling cycle profiles of PKNPs. (b) Absorption spectra of PKNPs before and after irradiation for 1 h.

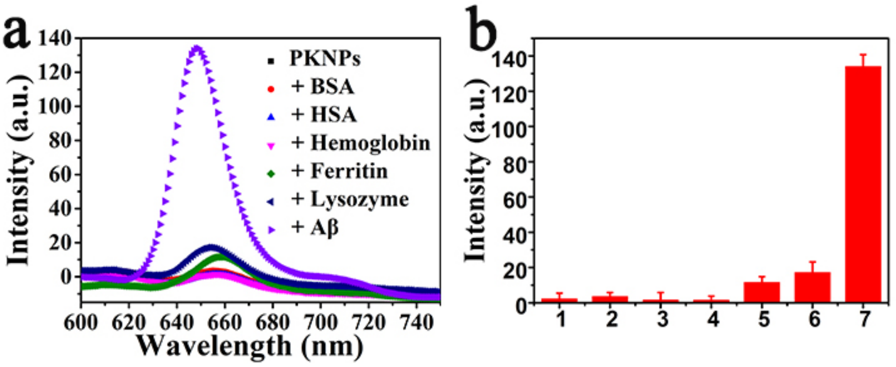

Figure S8. Fluorescence intensity responses of PKNPs to various proteins in water. 1 Blank; 2 Bull Serum Albumin (BSA); 3 Human Serum Albumin (HSA); 4 Hemoglobin; 5 Ferritin; 6 Lysozyme; 7 Aβ

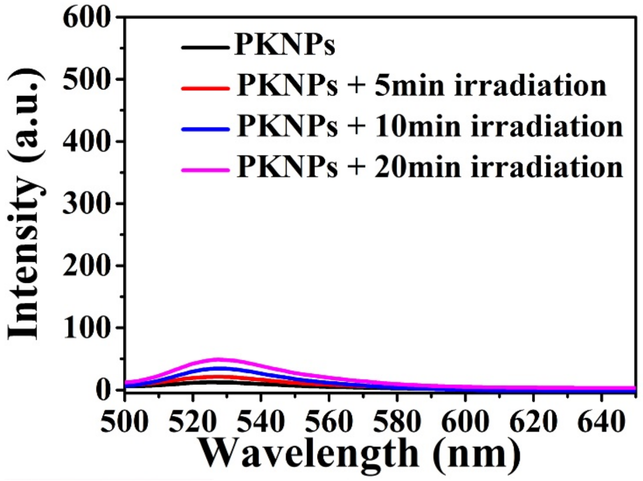

Figure S9. The ROS generation of PKNPs (0.2 mg mL^{-1}) irradiated for different time, using 2,7-dichlorofluorescin diacetate (DCFH-DA, 10 μM) as a probe.

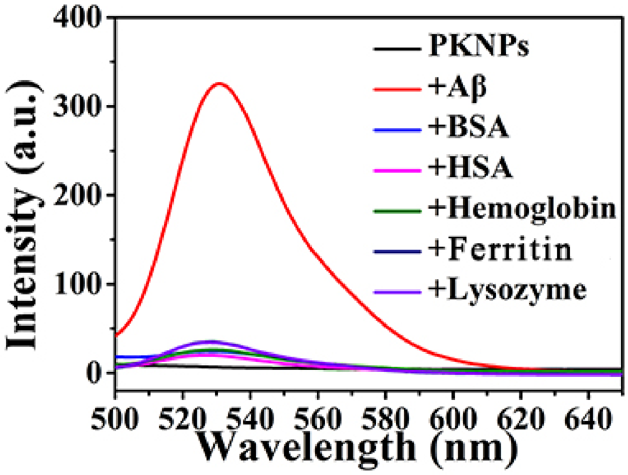

Figure S10. ROS generation of PKNPs (0.2 mg mL^{-1}) in the presence of various other proteins: 1 Blank; 2 Aβ; 3 BSA; 4 HSA; 5 Hemoglobin; 6 Ferritin; 7 Lysozyme; under laser irradiation for 10 min.

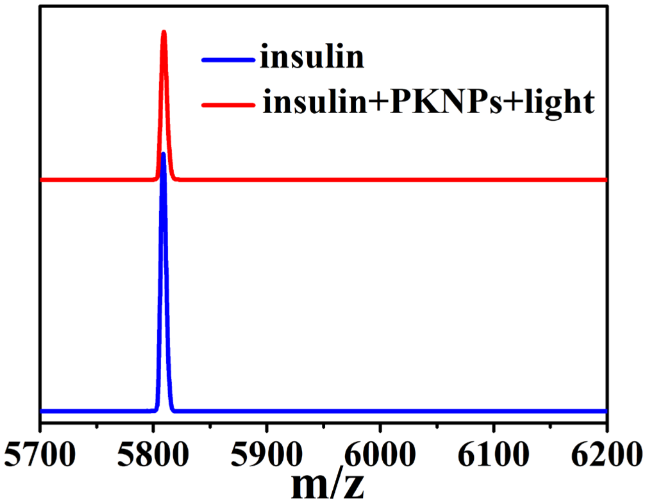

Figure S11. The mass spectra of insulin and insulin incubated with PKNPs under laser excitation.

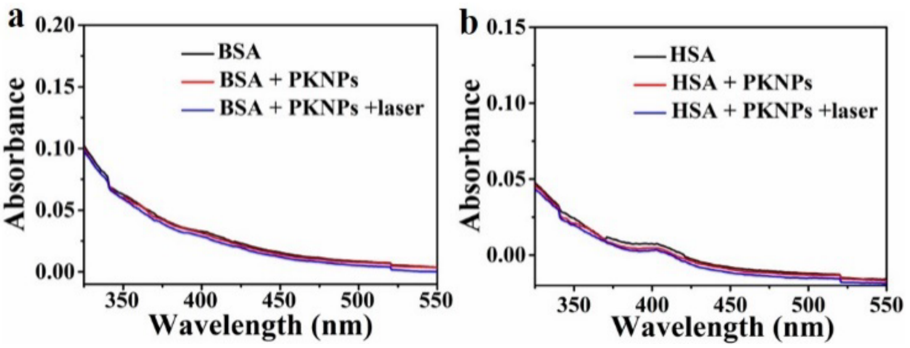

Figure S12. DNPH assay detect carbonyl content in BSA(a) samples and HSA(b) samples.

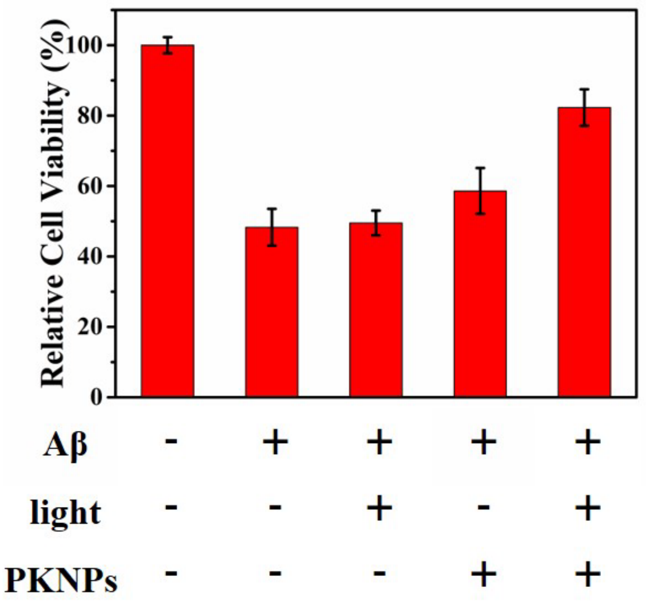

Figure S13. Protection effects of PKNPs (0.2 mg mL^{-1}) under irradiation on Aβ-induced cytotoxicity to PC-12 cells.

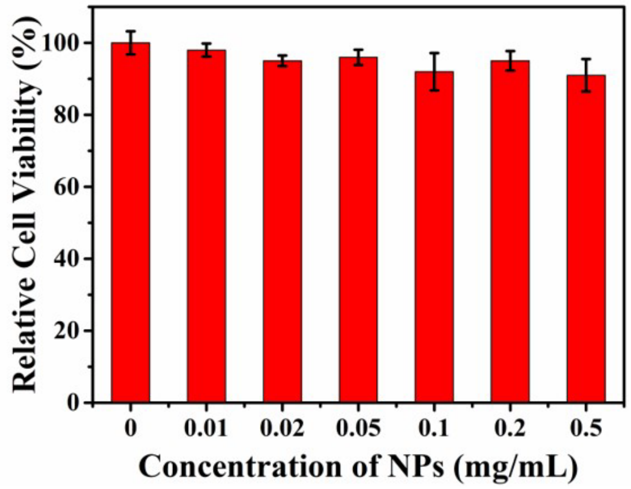

Figure S14. PC-12 cells viability in the presence of different concentration of PKNPs.

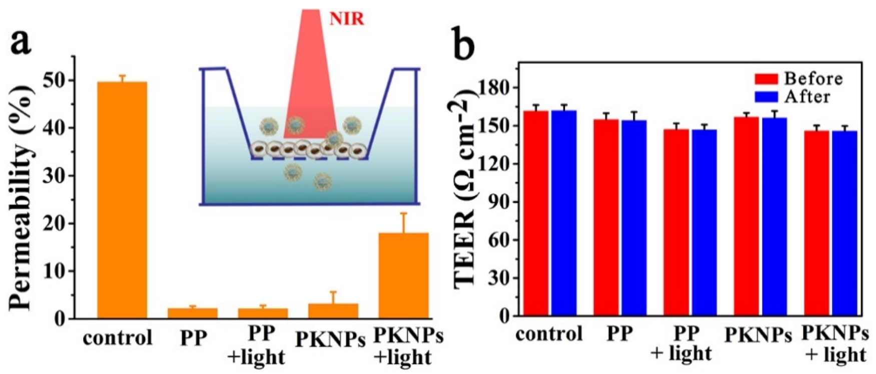

Figure S15. (a) The *in vitro* BBB crossing ability of PKNPs promoted by photothermal effect. Insert: schematic illustration of *in vitro* BBB model. Compared to the control

group (without bEnd.3 cell monolayer), the transport ratio of porphyrin and PKNPs were 2.12% and 3.09%, respectively. This indicates the successful fabrication of the *in vitro* BBB model and the transportation of substrates was inhibited. (b) The TEER values of the bEnd.3 monolayer were measured before and after the indicated treatments.

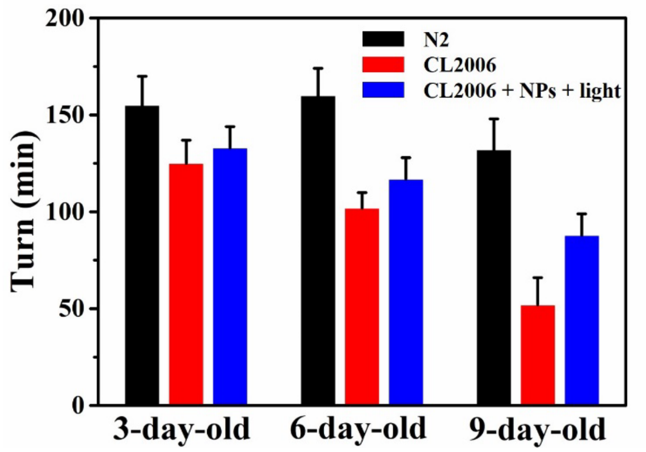

Figure S16. PKNPs improved the motility of CL2006 strain. Quantification of the worm movement in M9 buffer (turns per minute). N2, CL2006 and PKNPs treated CL2006 strains were 3, 6 or 9 days old.
